# The role of attachment styles in regulating the effects of dopamine on the behavior of salespersons

**DOI:** 10.3389/fnhum.2014.00032

**Published:** 2014-02-04

**Authors:** Willem Verbeke, Richard P. Bagozzi, Wouter E. van den Berg

**Affiliations:** ^1^Department of Business Economics, Erasmus School of EconomicsRotterdam, Netherlands; ^2^Ross School of Business, University of MichiganAnn Arbor, MI, USA

**Keywords:** attachment styles, *DRD2*, *DRD4*, customer orientation, sales professionals

## Abstract

Two classic strategic orientations have been found to pervade the behavior of modern salespersons: a sales orientation (SO) where salespersons use deception or guile to get customers to buy even if they do not need a product, and a customer orientation (CO) where salespersons first attempt to discover the customer's needs and adjust their product and selling approach to meet those needs. Study 1 replicates recent research and finds that the Taq A1 variant of the *DRD2* gene is not related to either sales or CO, whereas the 7-repeat variant of the *DRD4* gene is related to CO but not SO. Study 2 investigates gene × phenotype explanations of orientation of salespersons, drawing upon recent research in molecular genetics and biological/psychological attachment theory. The findings show that attachment style regulates the effects of *DRD2* on CO, such that greater avoidant attachment styles lead to higher CO for persons with the A2/A2 variant but neither the A1/A2 nor A1/A1 variants. Likewise, attachment style regulates the effects of *DRD4* on CO, such that greater avoidant attachment styles lead to higher CO for persons with the 7-repeat variant but not other variants. No effects were found on a SO, and secure and anxious attachment styles did not function as moderators.

## Introduction

Organizations are especially interesting social environments as they differ from everyday social groups such as found in family life, friendship, or hobby clubs. Within organizations, people undertake both long and short-term strategies to fit into their group and interact with others outside their group to meet the needs of their organization. Consistent with the emerging organizational cognitive neuroscience (OCN) framework (Senior et al., 2011), we seek to understand the biological processes—hard-wired neurological and endocrine processes conserved over millions of years in different species—that might help us understand how people operate in organizations, particularly those whose job requires them to deal with others outside their organization to meet their organization's mission. Specifically, we seek to explain the strategic orientation that salespersons take in their relationship with customers. Two fundamental, recently studied orientations are the sales orientation (SO) and customer orientation (CO) (Bagozzi et al., 2012). A SO involves the use of deception and guile by a salesperson to get customers to buy even if they do not need a product. A CO characterizes a salesperson's attempts to first discover the customer's needs and then adjust their product and selling approach to meet those needs. Sometimes the terms hard and soft selling are used to describe these orientations, where the latter generally leads to long-term relationships, whereas the former, given its one-sided exploitive nature, is typically short-lived.

Hard-wired neurological and endocrine processes, which undergird phenotypical selling and COs, provide ultimate explanations that define evolutionary fit outcomes. In developing our hypotheses and interpreting findings, which entail cross-level gene and phenotype descriptions, we draw upon molecular genetics research to ground our studies. Our approach is guided by two aims recently recommended in the literature, namely, (1) to replicate recent findings so as to show the relevance of candidate genes and set up the need to explore gene-phenotype interactions to explain strategic orientations of salespersons on the job (Munafò et al., 2008), and (2) to give special attention to definition and measurement of explanatory phenotypes and develop a theory accounting for how they moderate the effects of candidate genes on strategic orientations (Munafò et al., 2008).

Originally introduced in 1982 (Saxe and Weitz, 1982), the concepts of sales and COs and their measurement have found currency across many studies, where more than 30,000 salespeople have been investigated (Franke and Park, 2006). Nearly all of this research has been conducted at the psychological level of investigation, with self-reports as measures of independent and dependent variables. The sole exception appeared in a recent study by Bagozzi et al. (2012) (Study 2), where the *DRD2* A1 was found to be marginally associated with a SO (*p* = 0.07), and the *DRD4* 7R^+^ allele was found to be significantly associated with a CO (*p* = 0.04). The rationale for the former finding was that salespeople carrying the A1 variant should have a reduced response to dopamine, seek greater stimulation, and favor greater immediate gratification than carriers of the other variants, and therefore should be inclined to press customers into yielding without fully taking into account their needs. In contrast, the rationale for the latter finding was that salespeople carrying the 7R^+^ variant should be more curious and open to opportunity recognition, greater risk takers, and more inclined to search for unique needs of customers and put greater effort into finding and constructing a mutually beneficial match between buyer and seller.

A shortcoming of the study by Bagozzi et al. (2012) is that finding the main effects of candidate genes might occur by chance and reflect a false-positive outcome. To guard against prematurely placing too much credence on the findings in Bagozzi et al. (2012), it would be advisable to conduct replications on different subjects operating in different organizational environments. Further, discovery of the effects for individual candidate genes may be unrealistic in that factors other than genes may be of equal or greater importance or may be conditional on when and how genes function, if they function at all, in real-world job environments under naturalistic conditions. Therefore, our second aim is to develop a meaningful phenotype to explore a plausible gene × phenotype interaction effect on salesperson job orientation in the field. The phenotype chosen was the biological/psychological theory of attachment.

The OCN perspective seeks to uncover the role of higher-order psychological concepts in translational research by explicating hard-wired biological mechanisms and in doing so deepen and even change the measurement and functioning of these concepts (Senior et al., 2011). The challenge with developing strong hypotheses is that most studies in genetics are more on patients and less on healthy people, let alone people who operate in professional settings. In this regard, the *DRD2* (“reward or reinforcement gene”) and *DRD4* (“impulsive gene”) are known as risk genes, meaning that they are linked with such non-desirable phenotypes as addiction or impulsivity (e.g., Noble, 2000; Eisenberg et al., 2007; Green et al., 2008). Given the differential sensitivity hypothesis, which suggests that in different environments a particular gene might have opposite effects (Belsky et al., 2009), carriers of certain alleles of the *DRD2* or *DRD4* might actually thrive in certain environments, rather than necessarily exhibit the risk factors associated with clinical populations. Such a perspective might help us make better predictions and lead to better understanding of phenotypes and their effects. In what follows we explore the pathways in which *DRD2* and *DRD4* are expressed, and we investigate how polymorphisms of these genes regulate these pathways differently under the differential influence of the attachment phenotype.

Consequently, we investigate the moderating role of attachment, where we also examine a type of differential sensitivity and challenge the received view in the literature. Attachment theory arose out of clinical and cross-cultural research by Bowlby (1988) and Ainsworth (1991). A central claim is that young children develop stereotypical interpersonal styles because of relationships with early caregivers, typically the mother. Three distinct patterns tend to develop: anxious, avoidant, and secure. The anxious style is marked by the tendency to seek support from an attachment figure, to worry about being rejected, to harbor doubts about one's self-efficacy, to have low self-esteem, to crave attention and closeness, to feel vulnerable and helpless, and to possess a negative self-model, while being generally positive toward others because of a desire for support and protection. The avoidant style is characterized by a low need to feel close to others, a tendency to seek independence and self-reliance, and a propensity to focus on positive features of the self and downplay negative ones to build a positive self-model, while being dismissive or mistrustful of others. The secure style is distinguished by a positive self-image and relative openness and trust in relationships with others. Considerable evidence shows that attachment styles formed early in life persist to influence adult behavior (Mikulincer and Shaver, 2007).

Recent research with adults finds that the secure attachment style is the most functional across a wide variety of relationships. For example, consumer behavior research finds that people with secure, as opposed to anxious or avoidant, attachment styles form positive relationships and experience positive outcomes in service settings (e.g., Mende and Bolton, 2011). Research with employees in organizations shows that workers with avoidant and anxious attachment styles are less supportive in helping colleagues (Geller and Bamberger, 2009). We would argue, consistent with research with adults in family and romantic relationships (e.g., Mikulincer and Shaver, 2003, 2007), that the secure attachment style should be functional in everyday consumer behavior because consumers seek to find products that meet personal needs, and initial openness and trust when facing sellers should be conducive to meeting personal needs, whereas anxious or avoidant styles would interfere with the discovery of desired requisites. Likewise, within organizational boundaries, workers function best when cooperation and trust flourish and they strive to fit in and work together on common goals. Here a secure attachment style should promote such endeavors, whereas anxious and avoidant styles should interfere or lead to disharmony.

In contrast to research with consumers and workers *within* organizations, and opposite to predictions of attachment theory in romantic and family contexts, we argue that the secure attachment style will not be more functional than other attachment styles for salespersons, but rather the avoidant style will be most conducive to successful exchanges. This seeming paradox is based on the contingent role that the attachment phenotype plays in the unique context of business-to-business selling. Salespersons in such contexts function in decidedly *inter*-organizational environments where they venture away from the home organization to negotiate deals inside the buyer's organization. This not only weakens felt normative and peer pressure from the home organization, but exposes the seller to greater pressure from buyers in a more vulnerable setting, and leads to an interpersonal environment with more uncertainty, ambiguity, and tension than typically found in intra-organizational or personal relationships. Somewhat similar psychological tensions occur for ambassadors, diplomats, and inter-mediators in government and similar settings.

In a business-to-business context, informal norms and company policies by both seller and buyer firms typically caution, and even dictate and sanction, against the development of intimate or overly personal relationships (Anderson and Jap, 2005). Rather, buyer and seller are required to conform to professional rules of decorum and propriety. Codes of conduct and ethical guidelines govern personal involvement, fraternization, leaking of corporate information, and standards of behavior. Coupled with legal and moral issues concerning sexual harassment, bribery, kickbacks, and related topics, such work guidelines place real restrictions on the nature of social contact between sales representatives and buyers and color transactions. In addition, sales representatives operate as organization-boundary spanners and engage in such proactive behaviors as seeking new customers and making autonomous decisions when negotiating prices, especially in business-to-business contexts, all of which require sales representatives with an ability to behave efficaciously during interactions with customers (Crant, 1995).

These norms and expectations lead us to propose that avoidant styles are particularly suited for sales representatives in such relationships in business-to-business contexts. It is fruitful to conceive of attachment styles as working cognitive models on how one regards others and the self in social relationships in terms of the support one can give or get in times of need. Attachment styles are mental representations of person-person transactions that motivate one to seek protection or help from others in interpersonal relationships, to the extent that there is a threat or danger (Mikulincer and Shaver, 2003, 2007). Research shows that persons with avoidant attachment style prefer to hold a certain emotional distance from interaction partners to be able to keep the initiative and behave proactively (see Mikulincer and Shaver, 2003; Ein-Dor et al., 2010). Arguably, in common business-to-business settings, policies, and norms require that sales representatives uncover the needs of customers, offer solutions, and achieve commercial results. At the same time, persons with avoidant styles tend to be self-reliant (see Mikulincer and Shaver, 2003; Richards and Schat, 2011), which is a useful trait in sales representatives who operate in demanding inter-firm environments and are often physically away from both the home organization and its social support. Although some people are both high in avoidance and anxiety (termed in the literature, “fearful avoidance”), Mikulincer and Shaver (2003, p. 70) note that such persons are “less likely to arise in normal samples of college students and community adults” and are more common “in samples of abused or clinical samples.” Thus, the avoidant attachment style, where social anxiety is not a deficit, is consistent with modern characterizations of business relationships. Successful business-to-business sales representatives need to be sufficiently independent and detached, self-reliant, and not deterred by anticipatory anxiety to function well in such contexts (which tends to occur when representatives ask commitments of customers or when they have to close a deal; Vinchur et al., 1998; Richards and Schat, 2011). These conditions fit the avoidant attachment style well.

The secure attachment style is less conducive to the demands on sales representatives in business-to-business contexts. Researchers characterize the secure style as one where the person exhibits “comfort with closeness” and intimacy (Mikulincer and Shaver, 2003, p. 9). Such an orientation is not largely an asset in formal business relationships because buyers and sellers realize that there is potential for tension between the goals of buyer and seller organizations. Also, give and take are integral parts of the relationship, as both parties are required to meet the requisites of their home firms, which often do not fully coincide with the other firm's. Intimacy or comfort with closeness may even interfere with interactions in some business relationships. In addition, it is possible for employees to be too secure and not motivated as much by “the hunger to make a sale” or “the fear of failure,” whereas a person who is avoidant in orientation is more likely to be more motivated. The avoidant style places emphasis on business goals, not personal relationship ones, *per se*, although goals can be met mutually in business-to-business contexts, and thereby promoted largely when a CO vs. a SO is pursued. This is especially salient in inter-organization relationships.

The anxious attachment style also seems not to fit business-to-business settings as well as the avoidant style. Preoccupation with the fear of rejection or failure to make a sale, or “a strong need for closeness, [and] worries about relationships,” as found for anxious attachment style persons (Mikulincer and Shaver, 2003, p. 69; see also Ein-Dor et al., 2010, p. 134), would seem to lead sales representatives to work too hard to elicit immediate support and even affection from customers, which draws attention away from exploring via conversation the needs of buyers and then presenting a commercially viable solution to meet those needs and close the sale. The avoidant style should entail less disruptive and more realistic coping with fear or anxiety (e.g., Ein-Dor et al., 2010, p. 134; Richards and Schat, 2011).

The avoidant attachment style thus seems to strike a balance between the secure and anxious styles. To the extent that avoidant attached salespeople remain self-confident, they should abstain from relying too much on trust in others, meaning that they will retain a certain amount of self-reliance, spontaneity, and initiative to make sure customers understand offers and respond accordingly. The avoidant attachment style salesperson is therefore neither too secure nor too anxious but rather reflects a realization that selling to business customers is more rooted in a rational or professional relationship than a personal one *per se*.

In sum, we hypothesize that the avoidant attachment style, but not the anxious or secure, should function as the best moderator of the effects of the *DRD2* and *DRD4* genes on CO. How this happens also invokes differential sensitivity.

## Genetic study 1

The two genes, *DRD2* and *DRD4*, although often perceived as risk genes, might turn out to be functional in a selling context (Goodman, 2008; Tripp and Wickens, 2009). Both genes code for receptors for dopamine (a catecholamine), which is known to modulate synaptic transmission, especially in the cortex and striatum (Tritsch and Sabatini, 2012). Specifically, *DRD2* is mainly expressed in the ventral striatum and thus affects instrumental learning and conditioning, whereas the *DRD4* is mostly expressed in the prefrontal cortex (PFC) and affects how people process information and engage in self-regulation. These mechanisms for dopamine (D) modulation are vast, operating in pre-synapsis neurotransmitter release (e.g., vesicular release machinery), in post-synapsis detection of neurotransmitter detection (e.g., modulating membrane insertion), and synaptic integration and excitability (e.g., modulating ion channels) (Tritsch and Sabatini, 2012). Therefore, as Green et al. (2008) suggest, it is too simplistic to relate a specific gene polymorphism to a specific region of the brain, given the huge connectivity between the brain nuclei but also the great complexity of neuromodulation. Rather than one or a small number of regions of the brain involved, it is more realistic to expect many regions to be engaged in a complex system of interactions.

Here we mainly focus on the differential roles of the D1-like (D1 and D5) and D2-like (D2, D3, and D4) receptors in the intercellular integration within post-synapse areas. The D2-like receptors compared to the D1-like type receptors have a higher affinity for dopamine (10 to 100-fold greater for the D3 and even more greater for the D4) (Tritsch and Sabatini, 2012). A key for both cognition and reward system functioning is the D1/D2 ratio (dual state model). Here, the D1 receptor plays a gating role by controlling the threshold of significance above which information must pass before it can be admitted to working memory (achieving stabilization), and the D2 signals the presence of information (mostly reward based information) that allows the PFC network to respond to this new information by updating its working memory system (achieving flexibility) (Seamans and Yang, 2004; Savitz et al., 2006). The D1/D2 ratio regulation implies that D1-like receptors are bound to *stimulatory* G proteins (hence called G protein-coupled receptors) that energize adenylyl cyclase, and this activates the production of cyclic adenosine monophosphate (cAMP), and thus activation of protein kinase A (PKA). PKA mediates the phosphorylation and regulates the function of a wide area of cellular substrates such as K+, Na+ and Ca+, glutamate, GABA receptors, and transcription factors. D2-like receptors bind to *inhibitory* G proteins that hinder adenylyl cyclase and thus reduce the production of cAMP, which prevents cAMP activation of PKA and also reduces N-methyl-D-aspartate (NMDA) receptor activation and GABA-ergic inhibition (Seamans and Yang, 2004; Tritsch and Sabatini, 2012).

Dopamine levels have an effect on the D1/D2 ratio, but this effect is different in the PFC (slow modulation) compared to the striatum (reinforcing brief activity), thus complicating the ability to make clear conjectures (Tripp and Wickens, 2009). The striatum and PFC are mutually interconnected, as well as to the dopamine system, and thus stimulation by dopamine affects both reward seeking and planning, which is why dopamine levels have an inverted U curve effect on cognitive performance; both low and high levels of dopamine fail to affect cognitive performance, but intermediate levels effect cognitive performance strongly. This is because the striatum is activated more intensely by dopamine and (due to its connection with the PFC) leads to reductions in flexibility of switching costs, at least under some conditions such as in planning (Aarts et al., 2011).

For cognitive processes, when dopamine levels are high (low), there is a higher (lower) D1/D2 ratio, which due to cAMP activation and its intracellular chain reaction affects the excitatory release of glutamate from pyramidal cells of the PFC. Consequently, there is stronger excitatory signaling and better inhibition of noise due to distraction in the environment (in other words, more focus occurs). Higher PFC activation also feedbacks back to the striatum and allows for better regulation of striatal impulses (needed for self-regulation and inhibition). However, higher dopamine levels in the striatum have a different effect: activation in the striatum helps a person respond flexibly to environmental cues, especially for what is desired (routines and wanting). However, when strongly activated, the striatum might predispose a person to respond inflexibly to the environment as routine responding takes over (Aarts et al., 2011). In short, strong striatum activation might compromise cognitive flexibility or raise switching costs. We expect that the two candidate genes (*DRD2* and *DRD4*) will affect the D1/D2 ratio and thus have an impact on cognitive and reward processes. Somewhat similar outcomes happen with the *COMT* gene where Met carriers experience lower ability of enzyme breakdown of dopamine, and thus dopamine levels remain high, and a higher D1/D2 ratio occurs resulting in greater cAMP activation, higher glutamate levels, and greater cognitive focus, at the cost of more rigid behavior.

The *DRD4* gene (D2-like), located on chromosome 11p15.5, codes for the dopamine D4 receptor and includes in exon III a 48-bp variable number of tandem repeats (VNTR) polymorphism, which contains 2–11 repeats. This VNTR is located in a region that encodes the supposed third cytoplasmic loop of the receptor that couples to inhibiting G proteins, which reduce the production of cAMP, and thus inhibits the chain reaction in the neuron (Wang et al., 2004; Barnes et al., 2011). Carriers of the *DRD4* 7^+^ repeat (7R^+^) variant of this polymorphism in the *DRD4* gene experience reduced ability to blunt cAMP signaling in neurons (Asghari et al., 1995; Oak et al., 2000), compared to 7R^−^ carriers (both in the pre- and post-synapsis), and thus are less able to play an inhibitory role, so undergo higher glutamate activation. Due to the fact that *DRD4* is mainly expressed in the PFC, there is more cognitive elaboration and higher alertness for what might be new. This leads to the following cognitive and behavioral effects: the dopamine system switches too quickly from a tonic to a phasic state (higher sensitivity to reward salience) (Grace, 1991), and this makes the person more open to experience; indeed Munafò et al. (2008) showed that carriers of the *DRD4* 7R^+^ were more likely to show approach-related personality traits (especially novelty-seeking). Carriers of the *DRD4* 7R^+^ are less able to maintain cognitive self-control than non-carriers and thus are more vulnerable to distracting information, which if occurring in a sales conversation might consist in lost information that is relevant, such as happens with non-verbal signals. Similarly, carriers of the *DRD4* 7R^+^ are less able to self-regulate and have difficulties post-poning gratification, making them vulnerable to committing more impulsive behaviors (Munafò et al., 2008).

Successful selling requires salespeople to look for opportunities displayed implicitly in interpersonal encounters (e.g., being sensitive to implicit meaning and non-verbal communication) and explicitly by customers (e.g., voicing needs, objections). Salespeople who are carriers of the *DRD4* 7R^+^ might be more likely to respond to these changes and thus better sense opportunities than non-carriers.

The *DRD2* gene, located on chromosome 11q22-q23 (region rs 180049), codes for the dopamine receptor D2, and includes exon 8 of the ANKK1 gene (Ritchie and Noble, 2003). *DRD2* is especially active in the ventral striatum, and it is the most widely expressed D receptor in the brain (Tritsch and Sabatini, 2012). Carriers of the *DRD2* Taq A1 experience a reduction in both pre and post-synaptic D2 sites, which results in increased dopamine release. More dopamine means that there is a greater activation of neurons in the striatum (Laakso et al., 2005). As dopamine levels rise, so will activation of the striatum (the D1/D2 ratio changes accordingly, and the consequent intracellular cascade will occur). Due to the connection with the PFC, this might affect flexibility in cognitive tasks and produce a concave U effect. Optimal levels of dopamine might result in optimal cognitive performance, but too much dopamine results in lower cognitive performance. For example, Stelzel et al. (2010) found that carriers of *DRD2* Taq A1, were less proficient in adjusting their behavior based on feedback about earlier performance (but not when they engaged in a novel cognitive task). In addition, because the striatum (especially the NAcc) has the most D2-like receptors, there is also a higher probability that carriers have greater wanting and reward dependency (Trifilieff et al., 2013). Thus, they might be more motivated and willing to put pressure on customers due to their stronger wanting. Considering the facets of a SO described above, carriers of the *DRD2* Taq A1 might engage more frequently in a SO.

## Materials and methods study 1

### Subjects

A total of 64 salespeople, all working in business-to-business environments, were asked to participate in a study involving DNA analysis. They came from the following industries: 4% came from automotive, 3% from food and beverage, 15% from banking, 3% from utilities, 9% from manufacturing, 23% from professional services, 7% from pharmaceuticals, 2% from telecom, 5% from logistics, 20% from IT, 3% from retailing, and 6% from other industries. Respondents answered an online questionnaire containing CO and SO questions from the SOCO scale (Saxe and Weitz, 1982), identical to those used in the study by Bagozzi et al. (2012) (see Table 1). The response format was a 7-point disagree-agree Likert format. However, one item from the CO and two items from the SO were deleted because they loaded too low on their respective factors, based on exploratory factor analysis. Nevertheless, since one aim of our study is to replicate the original findings of Bagozzi et al. (2012), we will report results for the SO and CO scores on the scales from the current study, as well as the original scales as used by Bagozzi et al. (2012). The alpha of the (4-item) CO scale from this study was 0.71 (5-item Bagozzi et al., scale = 0.60). The alpha of the (3-item) SO scale was 0.76 (5-item Bagozzi et al., scale = 0.82).

**Table 1:** **Customer orientation and sales orientation scales (see Bagozzi et al., 2012)**.

**CUSTOMER ORIENTATION (CO)**	
1	I try to get customers to discuss their needs with me.
2	I try to find out what kind of product would be most helpful to a customer.^*^
3	I try to bring a customer with a problem together with a product that helps him solve the problem.
4	I try to give customers an accurate expectation of what the product will do for them.
5	I try to figure out what a customer's needs are.
**SALES ORIENTATION (SO)**	
1	I try to sell a customer all I can convince him to buy, even if I think it is more than a wise customer would buy.
2	I try to sell as much as I can rather than satisfy a customer.
3	If I am not sure a product is right for a customer, I will still apply pressure to get him to buy.^*^
4	I paint too rosy a picture of my products, to make them sound as good as possible.^*^
5	It is necessary to stretch the truth in describing a product to a customer.

* These items had low factor loadings, so all analysis were done twice: once with the original full scales above, and one with the full scales above, and once with the full scales with these items removed (see Tables 2, 3).

### Procedures and statistical analyses

We followed recommended practice to gather DNA data and analysis, and allele frequencies analysis using the Hardy–Weinberg Equilibrium. We use parametric *t*-tests for tests of equality of means on the CO scale and SO scale and *DRD2*/*DRD4* polymorphisms of participants.

## Results

Tables 2, 3 present the findings. The results for DRD2 show that neither CO (*t* = −0.69, *p* = 0.91; *t* = −0.85; *p* = 0.87) nor SO (*t* = −0.31, *p* = 0.77; *t* = –0.38; *p* = 0.70), differ significantly between the A1 and no-A1 variants. By contrast, for *DRD4*, 7R^+^ carriers have significantly higher means than non-carriers on CO (*t* = 2.37, *p* = 0.02; *t* = 2.60, *p* = 0.01), but no differences were found on SO (*t* = −0.11, *p* = 0.91; *t* = −0.50; *p* = 0.62).

**Table 2:** **DRD2 Taq A1 *t*-tests for equality of means**.

	**Group**	**Mean**	***t*-test (two-sided)**	***p*-value**
Customer orientation	No A1	6.33	−0.69	0.91
	A1	6.42		
Customer orientation (Bagozzi et al., 2012)	No A1	6.15	−0.85	0.87
	A1	6.26		
Sales orientation	No A1	5.33	−0.31	0.77
	A1	5.42		
Sales orientation (Bagozzi et al., 2012)	No A1	5.42	−0.38	0.70
	A1	5.31		

**Table 3:** **DRD4 48 bp VNTR *t*-tests for equality of means**.

	**Group**	**Mean**	***t*-test (two-sided)*^a^***	***p*-value**
Customer orientation	NO 7R	6.26	**−2.37**	**0.02**
	7R	6.59		
Customer orientation (Bagozzi et al., 2012)	No 7R	6.09	**−2.60**	**0.01**
	7R	6.42		
Sales orientation	No 7R	5.35	−0.11	0.9
	7R	5.38		
Sales orientation (Bagozzi et al., 2012)	No 7R	5.34	−0.50	0.62
	7R	5.49		

a Bold values are significant at a 5% significance level.

## Discussion

Molecular genetics has the potential to inform organizational theory about key phenotypes from a biological perspective. However, to have a significant impact both in predicting and understanding behavioral tendencies or traits, findings between variants of specific genes and phenotypes should be replicated using different independent samples. We replicated recent findings concerning the relationship between the *DRD4* and *DRD2* genes and CO and SO, respectively (Bagozzi et al., 2012). In particular, consistent with Bagozzi et al. (2012), we found that salespeople carrying the 7R^+^ variant of the *DRD4* gene have a higher propensity to engage in CO. In contrast, no relationship between the variants of the *DRD2* genes and SO was found. It must be noted, however, that in Bagozzi et al. (2012) the association between *DRD2* A1 and SO was only marginally significant (*p* = 0.07).

Our findings show a clear impact of genes on SO, which goes beyond the scope of behavioral genetics. We would like to point out that such replications of candidate gene studies are rare, and indeed failures to replicate are the norm (e.g., Seabrook and Avison, 2010). One group of researchers (Chanock et al., 2007, p. 655) characterizes the published literature in this regard as “a plethora of questionable genotype-phenotype associations, replication of which has often failed in independent studies.” The latter authors maintain that “the challenge will be to separate true associations from the blizzard of false positives attained through attempts to replicate positive findings in subsequent studies” (p. 655).

### Gene × environment interaction

Our aim in Study 2 is to develop a theoretical basis for hypothesizing the conditions for the effect of key dopamine genes in an organizational context by specifying a particular gene-environment (phenotype) interaction. Since the molecular genetics approach more directly reflects how the brain functions (in this case the dopamine system), we are able to better understand how actions are initiated and maintained. These molecular mechanisms potentially contribute to our understanding of the phenotype, since they offer an additional explanation as to how our brain influences our behavioral tendencies. Specifically, salespeople's curiosity and eagerness to understand customers' needs involve regulation of the dopamine system known to be involved in novelty-seeking and the related motivational processes reviewed above, as governed by attachment style individual differences.

Attachment systems imply double-sided mechanisms: people, when anxious, seek proximity with others but also need to *feel secure in relationships*, such that they can further broaden and build behavioral repertoires in different social environments. Attachment styles develop in young children (Van IJzendoorn, 1995) exploring their environment. They experience fear when confronted with challenging situations, and then seek proximity to attachment figures (such as parents) and, when present/supportive, secure attachment styles evolve such that children comfortably seek and feel support from significant others; especially oxytocin (OT) and dopamine are involved in this (see hereafter). Based on these experiences, children develop a secure *working model*, developing expectations for predicting future interactions (cognitive schemas) and believing that others will be available and respond empathically if necessary. Children can then co-regulate stress (achieving emotional comfort or “neuroception” of safety) and attain feelings of security, allowing them to broaden their social exploratory behaviors, develop a theory of mind (TOM), de-activate negative expectations and boost their coping skills, such as is reflected in better ability to not get distracted and to conduct cognitive reappraisal (Porges, 2003). Secure attached people also like to give comfort to others (e.g., Mikulincer and Shaver, 2003).

The pleasant feeling that comes from close interaction (social approach) occurs because when children are nurtured by their parents there is a modest increase in dopamine transmission in the NAcc, which activates dopamine receptors D1 and D2, and both influence affection and pleasure and help maintain social bonds. D1 and D2 have different effects on approaching behavior as they have contrasting effects in the intracellular mechanisms: D2-like receptors (expressed in neurons that project from the rostral shell of the nucleus accumbens to the ventral pallidum) are necessary for the formation of a pair bond. Specifically the D2 receptors are bound to inhibitory G proteins, which act to reduce the cAMP, which prevents PKA, and is associated with the facilitation of attachment (primary unconditional rewarding). D1 receptors are bound to stimulatory G proteins, which increases cAMP signaling, which in turn increases PKA, and results in reduced mating partner preferences, but especially reduces the seeking of new partners once a bond has been made. Key is that OT promotes the activation of inhibitory G proteins and down regulates the intracellular cAMP cascade. OT also enhances the hedonic value of social interactions by activating areas rich in dopamine receptors in especially the reward system (which includes the VTA, substantia nigra). OT changes how the dopamine system updates the outcome of actions; it reduces the feelings of risk (reduction in amygdala activation), and this motivates people to undertake social interactions and experience them as intrinsically rewarding. In other words, for many people, especially stable-attached persons, social interaction with significant others is intrinsically rewarding.

There is now evidence that secure interactions entail long-term changes in the brain: secure attached people have greater gray matter reward volume in the reward network and interconnected regions such as hypothalamus or orbito frontal cortex (OFC) (e.g., the ventral striatum is differentially activated in secure mothers when they see their own babies smiling or crying, Strathearn et al., 2009). In addition, secure mothers also experience increased gray matter volume in the amygdala, the longer the post-partum period; in other words, it shows that they have a greater affective vigilance for their own children compared to other children. Secure mothers also have greater gray matter volume in areas related to TOM processes, such as the PFC, STS, and fusiform gyrus, and higher BOLD (blood-oxygen-level dependent) signal responses when hearing babies, which shows that as they interact with people they constantly improve their TOM network.

When attachment figures are not reliably available or supportive (e.g., caregivers behave unpredictably or do not provide support), a healthy sense of security is not attained, and secondary strategies of affect regulation come into play. Two internal working models emerge: avoidant and anxious.

Avoidant people do not have a healthy approaching system and have reduced, or lack, reward-related activity during positive social situations; e.g., avoidant attached individuals rate positive social information as less arousing (e.g., avoidant mothers had low activation of the ventral striatum and VTA) or do not experience positive social interaction as intrinsically rewarding compared to secure mothers, as they deactivate the attachment system and therefore do not seek to approach people (Vrtička and Vuilleumier, 2012, p. 6). Avoidant people are more concerned with self-preservation, have a positive self-model, show distrust to a partner's goodwill, and strive to maintain independence. Strong self-reliance often develops. Besides experiencing relatively low feelings of pleasure in social interaction, avoidant attached people may exhibit ill-functioning emotional coping styles: avoidant attached people de-emphasize threats and tend to cope without help or support from others; e.g., when rejected they have a decreased activation of the anterior insula and dACC (DeWall et al., 2012), which indicates a blunted response to social negative contexts (or a lower need to feel included). The problem is that this blunting might not work when pressure is high. For example, Vrtička et al. (2012) show that when emotional regulation strategies are constrained, avoidant attached persons have higher amygdala responses to emotional stimuli.

Anxious people develop vigilance reactions: they hyperactivate the attachment system when stress occurs resulting in an inability to handle threats autonomously. Anxious people tend to exaggerate threats. For example, Vrtička et al. (2008) show that the amygdala was selectively activated when angry faces were presented as negative feedback after giving incorrect responses; this leads to heightened distress and higher emotionality. This amygdala activation shows that anxious persons experience heightened distress in situations of personal failure or social disapproval. Equally, when people are excluded from others in the Cyberball paradigm, they show increased activation of the anterior insula and dAAC, which means that they are sensitive to rejection (Eisenberger et al., 2003). They become very emotional, and despite feeling that others are inconsistent and not trustworthy, they attempt to gain protection and support. Anxious people also worry that partners will not be available in times of need and attempt to gain partner attention, care, or even love. Feelings of intense dependence and clinginess may emerge.

While most research shows that insecure people might not be strong in relationship building, there is now evidence from animal research and human research in organizations that insecure attached agents are actually very productive to fit. Beery and Francis (2011) show that rats when raised in insecure conditions (low licking and grooming) actually performed better on individual cognitive tasks than rats raised in secure conditions (high licking and grooming). In addition, school children with parents who did not look after them well, actually helped children in school better than children raised with parents who cared well for them (Obradović et al., 2010). Therefore, we are now looking for different sorts of events to substantiate this.

Beery and Francis (2011) suggest that stressful experiences in mice do not inevitably lead to dysregulation of stress reactivity and that increases in stress reactivity (caused by early life stress due to poor maternal care) are not necessarily dysfunctional. Beery and Francis introduce the concept of stress inoculation, meaning that changes in the HPA axis and reward system to stress learned in early maternal care might actually be beneficial within certain contexts; e.g., rats subjected to stress conditions exhibited less emotionality (Levine, 1962) and demonstrated efficient neuro-endocrine responses. Confirming the effects of susceptibility to environmental influences, stress reactivity to environmental cues can lead to greater responsiveness to stimulating environments in certain contexts.

Ein-Dor et al. (2010) speak about the paradox of attachment, by which they mean that many insecure people can actually perform well at certain tasks. Using an experimental design in which fire suddenly broke out, Ein-Dor et al. found that anxious people first noted the fire, whereas avoidant people were the first to take flight, and secure people followed the avoidant attached people in fleeing. Hence, there is evidence for concluding that in certain situations insecure attached persons might perform well and outperform secure attached persons.

### Hypotheses

#### DRD2 moderation

We propose that the effects of variants of the *DRD2* dopamine receptor gene on CO will depend on the degree of avoidance attachment style. Specifically, we hypothesize the greater the avoidance attachment style, the greater the CO for carriers of the A2, A2 allele but not either the A1, A1 or A1, A2 alleles. Carriers of the A2, A2 allele vs. the other alleles are less distracted by intrusive or anxious thoughts (stemming from rumination and anticipated rejection by customers or worry that the customer will think that one is unattractive or less competent) and should therefore be more focused on the needs of customers, listen attentively, and respond to changing interpersonal give and take. In contrast, carriers of the A1, A1, or A1, A2 allele should be more rigid in their thinking and engage inflexibly in stereotypical behavior patterns (van Holstein et al., 2011). In other words, expected higher switching costs for carriers of the A2, A2 allele, compared to carriers of the A1, A2 or A1, A2 alleles, should be associated with greater focus and persistence, when salespersons interact with customers, which fosters the ability to adjust product/service offerings and one's communications to customers. Carriers of the A1, A1 and A1, A2 alleles, compared to carriers of the A2, A2 allele, should not only be more susceptible to distraction but also more impatient and unfocused.

#### DRD4 moderation

The *DRD4* dopamine receptor gene exists in variants that affect receptor activation by the dopamine neurotransmitter. Specifically, carriers of the 7R allele (7R^+^), vs. non-carriers, have been found to engage in more risk taking (Dreber et al., 2009), novelty-seeking (e.g., Ebstein et al., 1996; cf., Munafò et al., 2008), and opportunity recognition during customer interactions (see Study 1 in the current paper; Bagozzi et al., 2012). Work to date has focused largely on the main effects of these gene variants, but we examine their modulating effects on the impact of the avoidant attachment style on CO. Consequently, we expect an interaction effect: the avoidant attachment style will lead to greater CO in salespeople with the 7R^+^ allele but not for salespeople without it. The rationale is that for sales representatives with the 7R^+^ allele, the greater the inclination to be open to taking risks and pursuing new opportunities, the more an avoidant attachment style will lead to a strong CO. Again, we argue that the avoidant attachment style is manifest in an ability to remain efficacious and goal driven when discussing customer needs, and present appropriate solutions without allowing feelings of rejection to intrude detrimentally and adversely affect one's efforts (see findings in the psychology literature on “suppressing distress-related thoughts,” Ein-Dor et al., 2010, p. 134).

## Materials and methods study 2

### Subjects

Hypotheses were tested on a sample of 73 sales representatives who volunteered for a study of the role of biomarkers in professional relationships. Participants provided written informed consent, and the study was approved by the local research ethics committee. Participants were not told about the aim of the study at the start but were debriefed after completion of the study. All participated in post-graduate executive education programs. All were business-to-business salespeople selling financial services, trucks, IT services, insurance, pharmaceutical drugs, or consulting services. These selling positions require more thorough and repetitive conversations with customers compared to sales interactions with consumers where impulsive buying and transactions play a more important role (e.g., retail sales; door-to-door selling). All were Caucasian, 87% men, 13% women, 49% had a university degree and the rest vocational school diplomas. The average level of selling experience was 6.8 years. All participants donated saliva so that their DNA could be analyzed for the two candidate genes, *DRD4* and *DRD2*.

### Procedure

Attachment styles were measured with 12 7-point “does not describe me at all” to “describes me very well” end-points, and “describes me moderately well” as a mid-point (see Table 4). These items were adapted from Professor Phillip R. Shaver's latest scale, which he kindly provided^1^. This scale is based on the original in Hazan and Shaver (1987), which was revised by Collins and Read (1990). Note that there are six items for anxious attachment, three for avoidant, and three for secure.

**Table 4:** **Attachment style scales**.

**ANXIOUS**	
1	I worry that others won't care about me as much as I care about them.
2	My desire to be very close sometimes scares people away.
3	I need a lot of reassurance that I am loved by my partner.
4	I do not often worry about being abandoned.
5	I find that my close relationships don't want to get as close as I would like.
6	I get frustrated if partners are not available when I need them.
**AVOIDANT**	
7	I want to get close to others, but I keep pulling back.
8	I am nervous when partners get too close to me.
9	I try to avoid getting too close to others.
**SECURE**	
10	I usually discuss my problems and concerns with my partner.
11	It helps to turn to my romantic partner in times of need.
12	I turn to my partner for many things, including comfort and reassurance.

CO was measured with 5 7-point disagree-agree items with the same format used as for the attachment style items. This scale was developed by Bagozzi et al. (2012) as a subset of Saxe and Weitz's (1982) original scale. Table 1 shows the items.

## Results

Two items from the attachment scale were deleted because they loaded too low on their respective factors, based on an exploratory factor analysis (items 6 and 10). Cronbach's alpha reliabilities for the subscales were 0.69 for anxious, 0.81 for avoidant, and 0.67 (*r* = 0.51) for secure. Because all three factors were uncorrelated with each other, and empirical under identification occurred, we could not run a confirmatory factor analysis (CFA) for all three subscales together. A CFA for the anxious and avoidant subscales fit well: χ^2^_(19)_ = 17.65, *p* = 0.54, RMSEA = 0.00, NNFI = 1.01, CFI = 1.00, and SRMR = 0.076.

For the CO scale, the CFA model fit well: χ^2^_(5)_ = 4.65, *p* = 0.44, RMSEA = 0.00, NNFI = 1.00, CFI = 1.00, and SRMR = 0.036. Cronbach's alpha was 0.77.

Regressions were done according to standard procedures: first, we added the main effects, then the interaction effect. Here we only report the significant main findings. As we have dichotomous and continuous independent variables, we followed Jaccard and Turrisi (2003) to analyze interaction effects and graphically display the findings (see Nieuwenhuis et al., 2011). For the *DRD2* analyses, the two regression equations are, with *DDR2* coded (A1, A1 and A1, A2) = 1 and A2, A2 = 0 in the first regression and the reverse for the second:

**Table:** 

Customer orientation =	5.986	+0.204	avoid	+0.138	*DRD2*	−0.248	avoid × *DRD2*
	(0.098)	(0.074)		(0.149)		(0.109)	
	61.35	2.75		0.930		−2.29	
Customerorientation =	6.124	−0.044	avoid	−0.138	*DRD2*	+0.248	avoid × *DRD2*
	(0.112)	(0.079)		(0.149)		(0.109)	
	54.68	−0.55		−0.93		2.29	

where standard errors are in parentheses and *t*-values appear below them. This model fit well: *F*_(3, 69)_ = 2.73, *p* = 0.05, *R*^2^ = 0.11.

**Figure 1:**
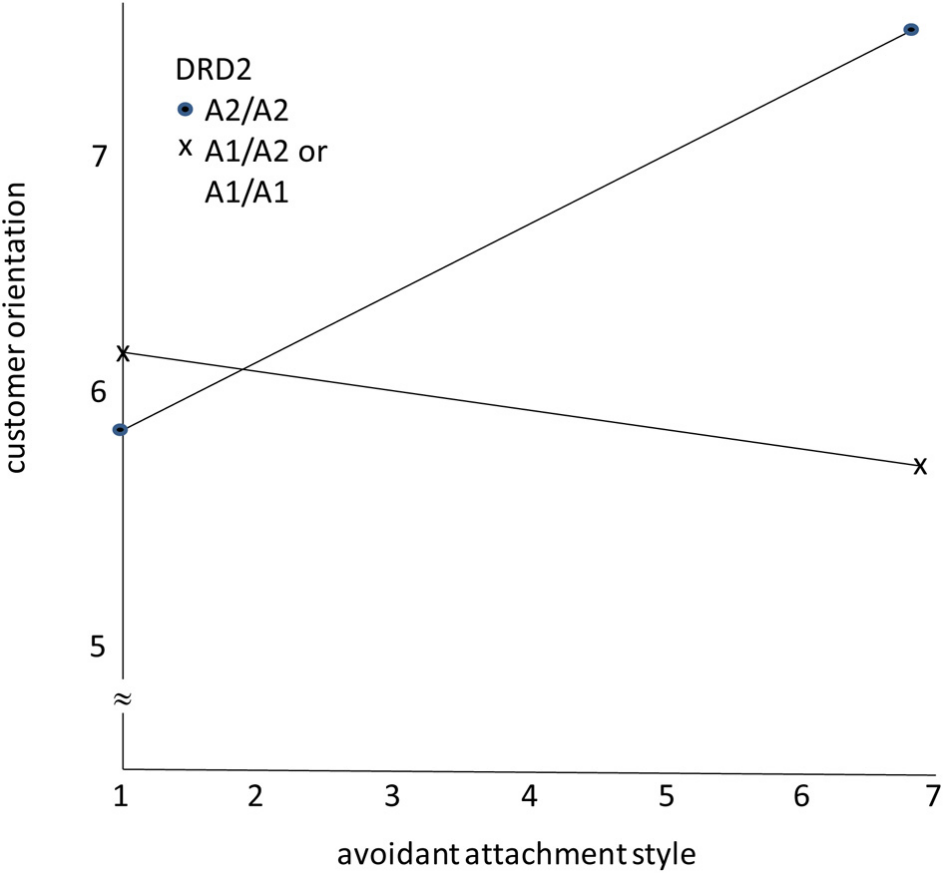
**The moderating role of DRD2 gene variants on the effects of avoidant attachment style on customer orientation**.

Figure 1 presents the results. As hypothesized, the avoidant attachment style has a positive effect on CO for sales representatives with the A2, A2 variant of the *DRD2* gene. For sales representatives with the A1, A1, and the A1, A2 variants of *DRD2*, the avoidant attachment style has little effect on CO, as predicted.

For the *DRD4* analyses, the two regression equations are, with *DRD4* coded 7R = 0 and 7R^+^ = 1 in the first regression and the reverse in the second regression:

**Table:** 

Customer orientation =	6.116	+0.038	avoid	+0.287	*DRD4*	−0.395	avoid × *DRD4*
	(0.084)	(0.057)		(0.174)		(0.166)	
	72.79	0.67		−1.64		2.38	
Customer orientation =	5.829	−0.433	avoid	−0.287	*DRD4*	+0.395	avoid × *DRD4*
	(0.153)	(0.155)		(0.174)		(0.166)	
	38.11	2.79		1.64		−2.38	

This model fit well: *F*_(3, 69)_ = 2.85, *p* = 0.04, *R*^2^ = 0.11.

**Figure 2:**
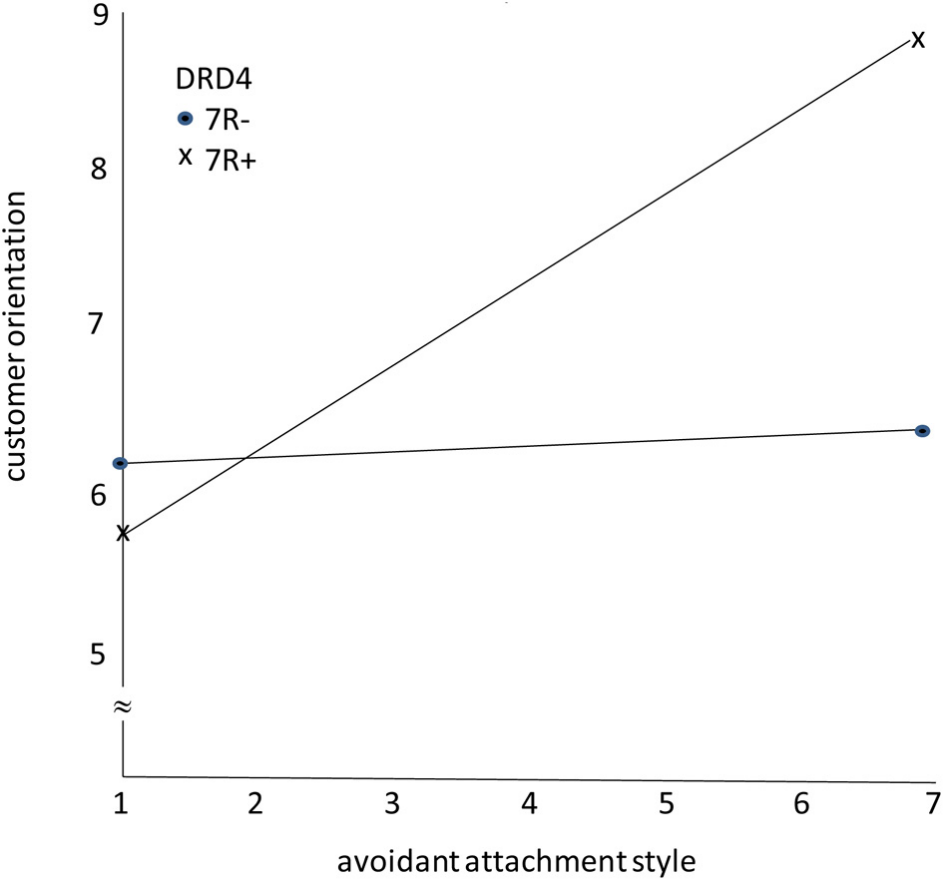
**The moderating role of DRD4 gene variants on the effects of avoidant attachment style on customer orientation**.

Figure 2 shows the findings. As predicted, the avoidant attachment style has a positive effect on CO for salespeople with the 7R^+^ variant of the *DRD4* gene. However, for salespeople with the 7R^−^ variant of the *DRD4* gene, the avoidant attachment style had no effect on CO, as expected.

To gain perspective, we also examined the interaction effects on CO of the anxious attachment style with *DRD2* and with *DRD4* polymorphisms, and the interaction effects on CO of the secure attachment style with *DRD2* and with *DRD4*. None of the interactions and none of the main effects were significant in the four regressions.

Also for perspective, we note that CO was not significantly correlated with the anxious attachment style (*r* = 0.16, ns), avoidance attachment style (*r* = 0.07, ns), secure attachment style (*r* = 0.11, ns), *DRD2* (*r* = 0.07, ns), or *DRD4* (*r* = 0.07, ns). Thus, CO was influenced only by the interactions of the avoidance attachment style with *DRD2* and with *DRD4* polymorphisms.

## Discussion

As we move into a biology-informed era in social research, researchers will benefit from scrutinizing such higher-order concepts as attitudes, personality traits, and work orientations using lower-order concepts from neuroscience (e.g., Becker et al., 2011; Senior et al., 2011) and molecular genetics. Whereas in our Study 1 we used insights from molecular genetics to replicate previous findings about the association between variations of two candidate genes, namely *DRD2* and *DRD4 (nature)*, in Study 2 we explored how gene activity is affected by interactions with the environment (*nurture*). We investigated this question because we believe that findings from such cross-level studies can enrich theory testing and knowledge development and guide practical decision-making by human resource managers. For customer boundary spanners, a meta-analysis by Ford et al. (1988) investigated how biographical and psychological variables compare in their effects on salesperson's success. Surprisingly, the results seemed to suggest that biographical information predicts performance better than psychological variables (see also Vinchur et al., 1998). Specifically, the findings showed that personal history and family background explained around 5% of the variance in performance and marital status accounted for less than 2%; in comparison, cognitive abilities explained less than 1% and vocational skills less than 1% of performance. Biographical variables, of course, beg the questions what in one's background influences behavior and what the underlying mechanisms are. The low levels of explained variance for both biographical and psychological variables suggest that the variables function poorly as main effects, and sound theories proposing interactions might be fruitful to explore in a person-by-situation exploration.

More specifically, two problems with such background variables can be identified. First, these variables can be thought to be one-step removed from the origin of salesperson behavior and serve as proxies at best for proximal psychological determinants of behavior. Second, the use of background variables in managerial decision-making risks the stigma of excessive intrusiveness, or even worse, the application of prejudice or profiling due to race, gender, or other categories.

In an effort to elucidate the interplay of *nature* and *nurture* on the etiology of SO, we examined how variants of the *DRD2* and *DRD4* genes moderate the effects of sales representative attachment styles on CO. The findings showed that the avoidant attachment style has a positive effect on CO for sales representatives carrying only *DRD2* A2 alleles, but no effect occur for sales representatives with at least one *DRD2* A1 allele. The avoidant attachment style has been shown to exhibit an orientation of emotional distance, yet a high degree of self-reliance, which seemingly fits expectations in inter-firm business relationships. However, whether, and to what extent, the avoidant style will influence CO apparently depends on the functioning of the dopamine system with regard to goal-directed, motivational, and reward-related behavior.

Carriers of the *DRD2* A1 allele exhibit reduced switching costs compared to carriers of only A2 alleles in intentional cognitive tasks (Stelzel et al., 2010). This should be manifest in greater task focus and persistence by the latter compared to the former, and greater sensitivity to task distracters and greater impatience for the former compared to the latter. The pattern of findings in Figure 1 is consistent with this interpretation, where we found that greater adherence to an avoidant attachment style leads to a stronger CO for sales representatives with the A2 alleles, whereas sales representatives with at least one A1 allele show no relationship between avoidant style and CO.

Furthermore, carriers of the *DRD4* 7R^+^ allele, vs. the 7R^−^ allele, have been shown to be greater risk takers and have a propensity to seek opportunities while interacting with customers. This, too, appears to regulate the effect of an avoidant attachment style on CO. We speculate that the tension occurring between the need to keep a certain amount of distance between self and customer, and the drive to seek new opportunities leads to a greater application of skills meeting (mutual) needs and greater chance of success.

Additionally, the present research also brings into focus the role in which insecure attachment styles (anxious and avoidant), as opposed to the secure attachment style, play in professional lives. In this regard, Ein-Dor et al. (2010) speak about the attachment paradox. Overall, researchers in psychology (e.g., Shaver and Brennan, 1992) have assumed that people with secure attachment styles fair better than those with insecure ones, with respect to building stable social relationships. The secure style is thought to promote stable relationships with others, because it is believed to increase fitness within the human species. However, when faced with vulnerable relationships or threatening situations, such as in many inter-firm selling contexts, people with an avoidant attachment style remain self-efficacious and goal driven, and maintain the initiative to seek innovative solutions (Ein-Dor et al., 2010). As Ein-Dor et al. speculate, avoidant attachment styles may be beneficial in certain situations. Our study shows that professional selling in business-to-business markets is such a context. Sales representatives are boundary spanners who work largely autonomously, explore the needs of customers, and shape the way customers view their own problems (Vinchur et al., 1998). They do so while maintaining a professional attitude in the face of conflicts of interest, misunderstandings, and customer resistance. In other words, whereas a secure attachment style might be best for in-group relationships, an avoidant style seems best for ingroup-outgroup relationships.

### Future research and practical implications

Our research paves the way for future discoveries. It would be productive to study different phenomena in organization behavior such as job attitudes, social identity, burnout and resilience, and motivation, and explore the role of genetics in combination with environmental factors. Such approaches are challenging, yet they might provide us with more insights into the concepts under study and their effects, which we exemplified in this study. Such insights also allow human resource managers to uncover what biological mechanisms are related to the (higher order) concepts they regularly use.

Elaborating on the study in this paper, we note that sales representatives do not always work alone but often in teams. Would sales teams of people who possess heterogeneous attachment styles function better than those with homogeneous styles? Such teams might contain people who seek psychological comfort (those with anxious attachment styles), sense competitive signals (those with anxious and avoidant attachment styles), and effectively implement interpersonal-change actions (especially those with avoidant attachment styles). As we studied the effects of attachment styles in interaction with genes, such questions are both difficult to ask and difficult to answer.

In terms of task-person fit, what attachment style should be employed by managers that supervise sales representatives with diverse attachment styles? Will managers with secure attachment styles, because they are perceived as open and trusting, attain better results, and can they bring both secure and insecure sales representatives together because they are inclined to promote cooperation, hence enhancing group or team formation and flexibility? Alternatively, could it be that managers with avoidant attachment styles empower their sales representatives because they do not seek unneeded or excessive closeness? Note that our findings showed that attachment styles interacted only with specific genes to influence COs. Holders of other genes might require different leadership strategies or better fit tasks other than boundary spanning roles.

Finally, attachment styles and people's genetic profile are stable and so tend to evoke automatic reactions or predictable tendencies in particular situations. Future research should study how sales representatives self-regulate such automatic tendencies and shape them into productive work orientations. For example, should firms make attachment styles part of awareness training? If attachment styles interact with genetic abilities, would such knowledge make sales representatives self-conscious of their genetic backgrounds and encourage or discourage adaptive behavior? Our findings invite researchers to explore the consequences of deeper, unconscious biological processes that shape human behavior in diverse organizational contexts.

Genetic data and measures of attachment style, if employed sensitively and applied ethically to hiring, training, and supervisory decisions along with other information, can provide more valid and fair criteria for management than reliance only on background information, interviews, and psychological tests. Of course, any use of such information must be based on validation of their effects on performances in any context, if Equal Employment Opportunity Commission Regulations and anti-discriminatory policies are to be met. Much remains to be done concerning our understanding of the role of genetic factors in organizational behavior. For example, more work is needed into how key genetic variables inter-relate with personality and situational constraints to influence behavior and outcomes. The pursuit of such ends promises to help us understand the “why” of behavior in organizations and provide policy insights.

### Limitations

One shortcoming of our research concerns the construct validity of our phenotype measures for CO, SO, and the three attachment styles. We acknowledge that full analysis of construct validity requires a multitrait, multimethod matrix investigation to assess convergent and discriminant validity. We did not conduct such a study, but some of the features of our approach suggest that construct validity may not be a significant problem. All our measures of variables were drawn from scales used before in a number of studies, thereby receiving some support for validity of measures in different research contexts with different samples. Second, all our measures achieved satisfactory reliabilities, and our factor analyses revealed that convergent and discriminant validity of measures were achieved, albeit with a monomethod approach. Future research could use confirmatory factor analysis in a multimethod design to better establish construct validity (Bagozzi, 2011).

We studied sales representatives to investigate the nature-nurture question related to molecular genetics in organizations. While this context provided initial answers, there are limitations.

First, one can argue that the sample sizes used in this study are small. However, we employed a hypothesis-driven approach, targeting only two genes and based on theory from biology and psychology, which reduces the need for large sample sizes required by exploratory searches across many genes. Importantly, we replicated findings presented by Bagozzi et al. (2012), regarding the association between carrying the *DRD4* 7R^+^ variant and the propensity to engage in customer-oriented selling. Convergent findings by two independent studies with regard to a specific genetic variant are rare in biological research and significantly contribute to the validity of the phenomena under study. Furthermore, the discovery of gene-environment interaction effects is also rarely recounted in the literature. Such interactions require the specification and test of unusual cross-level hypotheses and when found provide strong evidence for the mechanisms under research. In addition, while the costs of genetic profiling are becoming more feasible, such genetic studies compared to pencil and paper tests are difficult to implement.

Second, the application of molecular genetics research in organization theory and social research contexts would benefit from Genome Wide Association Studies (GWAS). This could uncover a small number of fundamental genes at work in the workplace. The following can be noted in this regard. First, as recommended by Senior et al. (2011) we selected genes for study that have already received some basic research efforts in areas of psychology relevant to our research. Thus, our inquiry was grounded in a specific, well-defined research tradition where in one sense our findings add to this body of knowledge. Second, GWAS require large sample sizes, because they test for up to one million genetic variants at the same time, introducing severe multiple-testing design and statistical issues, and thus significantly increasing the risk for false-positive findings. Finally, in order to build the large cohort that is required to give enough power for GWAS analyses, one needs to study heterogeneous samples, which in our case would mean studying people across many occupational settings and environments and making it difficult to draw conclusions pertaining to the specific work setting we investigated. Given the limited effect sizes that are typically observed in (candidate) gene studies, this might create too much noise in the sample to be able to arrive at valid genetic effects.

Third, we assumed that attachment styles are a reflection of environmental interactions, and therefore are a proxy of the influence of *nurture*, so to speak. However, attachment styles may have genetic association as well (e.g., Gillath et al., 2008). In addition, attachment styles were inferred from questionnaires in our studies, but more objective data could have been used; e.g., observations by clinicians or other experts.

Finally, we used an attachment style questionnaire tailored to how people experience general interpersonal relationships as adults. We could have developed a domain-specific attachment style measure tailored to the organizational context (e.g., Little et al., 2010). However, since we aimed to understand how environment and genes interact to influence behavior, we chose as our measure one that reflects the phenomenon under study in a way that functions during the critical window when one's neurobiological (stress) systems were shaped. This helps tie the findings for the adults under study to the early biological underpinnings and learning that produced the hypothesized consequences on the job.

### Conflict of interest statement

The authors declare that the research was conducted in the absence of any commercial or financial relationships that could be construed as a potential conflict of interest.

## Footnotes

^1^Personal communication with Professor Phillip R. Shaver, January 10, 2011.
